# How Uncertainty Bounds the Shape Index of Simple Cells

**DOI:** 10.1186/2190-8567-4-5

**Published:** 2014-04-17

**Authors:** D Barbieri, G Citti, A Sarti

**Affiliations:** 1 CAMS, EHESS/CNRS, 190-198, Avenue de France, 75244, Paris, France; 2 Dipartimento di Matematica, Università di Bologna, Piazza di Porta San Donato 5, 40126, Bologna, Italy

**Keywords:** Visual cortex, Uncertainty principle, Lie groups, Receptive profiles

## Abstract

We propose a theoretical motivation to quantify actual physiological features, such as the shape index distributions measured by Jones and Palmer in cats and by Ringach in macaque monkeys. We will adopt the uncertainty principle associated to the task of detection of position and orientation as the main tool to provide quantitative bounds on the family of simple cells concretely implemented in primary visual cortex.

**Mathematics Subject Classification (2000)2010:** 62P10, 43A32, 81R15.

## 1 Introduction

**Fig. 1:**
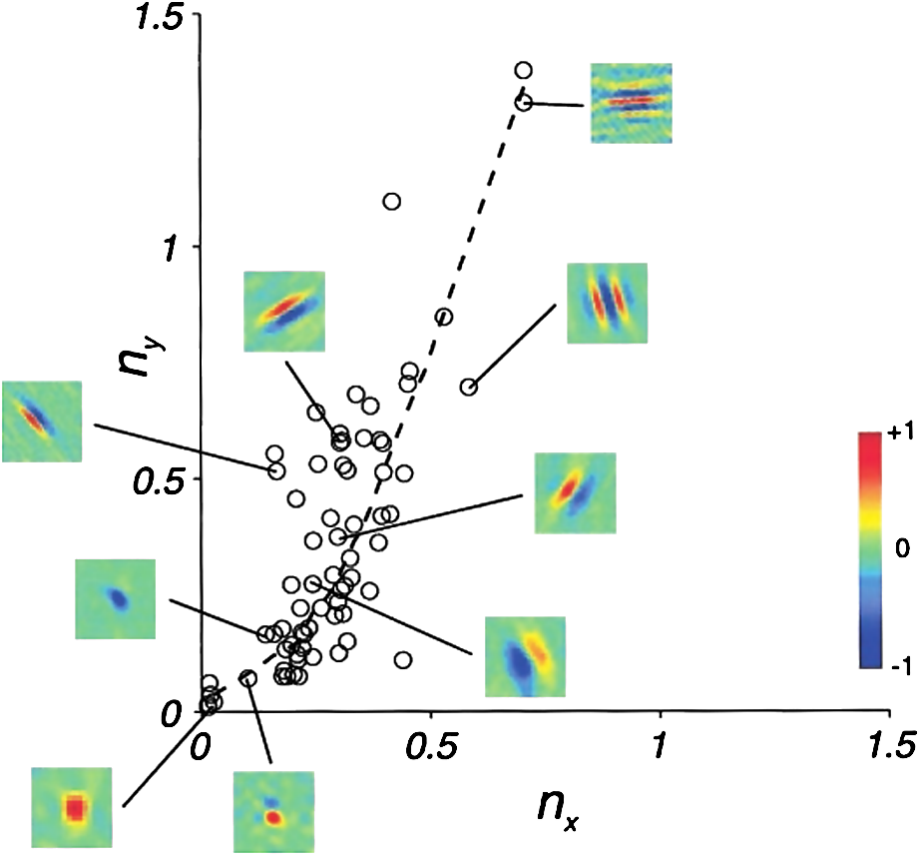
Distribution of receptive profiles in terms of their shapes. Figure extracted from [3]

One of the fundamental tasks performed by simple cells in primary visual cortex is that of detecting position and local orientation of a stimulus [1]. On the other hand, the functional behavior of simple cells as visual detectors is characterized in terms of standard linear filtering and with other so-called nonclassical behaviors [2]. We will concentrate on linear aspects, and consider classical receptive profiles modeled with a planar oscillation under a spatially localizing window. In [3], such receptive profiles were studied in terms of two dimensionless indexes of shape $\left(n_{x},n_{y}\right)$ corresponding to the product of the frequency of the oscillation and the sizes of the window in the direction of the oscillation and in the orthogonal one, showing that the distribution of such feature on V1 simple cells of macaque monkeys is confined to a specific region. This result is summarized in Fig. 1. Remarkably, the same confinement was found also in cats [4,5], and this suggests that this pattern can be associated with some criteria of optimality with respect to perceptive tasks. Notable proposals of such criteria were stated in terms of sparse coding in [6], already discussed in [3], and more recently in [7], or in terms of Bayesian learning [8]. 

In this paper, we will focus on the task of position and orientation detection, and propose theoretical motivations based on the uncertainty principle for the corresponding geometry to explain such confinement. In general, the uncertainty principle is indeed a tool that gives information on the possible localization of functions with respect to competing symmetries that in this case are those of the well-known group of translations and rotations of the Euclidean plane. The role of symmetries in the mechanisms of visual perception in V1 is a well recognized point [9-11], as well as the uncertainty principle was already invoked to explain relevant cortical morphologies [12,13]. Here, we will use such concepts to characterize the resolution that can be obtained with joint spatial and angular measurements, based on the localization properties of receptive profiles. In terms of such characterizations, we will deduce the bounds observed in Fig. 1 as the result of intrinsic notions of balance between joint measurements resolutions.

## 2 Receptive Profiles and Relevant Symmetries

We will assume isotropic Gaussian Gabor filters as a model for standard V1 simple cells classical receptive profiles, defined on the Euclidean image plane:

(1) $$\psi_{q,p}^{\sigma }(x)=\frac{1}{\sigma \sqrt{\pi }}e^{ip\cdot (x-q)}e^{-|x-q{|}^{2}/(2\sigma^{2})},\;x\in R^{2}$$

with parameters $q\in R^{2}$, $p\in R^{2}$, $\sigma \in R^{+}$. Each V1 simple cell is assumed to perform a linear filtering with a function shaped as in (1), so that it can be characterized by these parameters. Their mapping on the two dimensional cortical layers are referred to as cortical maps [14]. In particular, the centers *q* of receptive fields are in a so-called retinotopic correspondence on the cortex [1], while the size *σ* is in average larger at the periphery and smaller close to the fovea [15]. The frequency parameters *p* are generally considered in polar coordinates p=|p|(cosθ,sinθ), where |p| is called spatial frequency and the angle *θ* up to a factor of *π* is called preferred orientation, and their cortical maps are also well studied [11,13,16]. 

The family of functions (1) were proposed in [12] due to their optimal localization in space and frequency with respect to the classical Heisenberg uncertainty principle, and their fitness to model the linear behavior of simple cells was thoroughly tested [3]. We note, however, that here we are dealing with a simplified model of isotropic receptive fields, since as we will see this provides enough information for the present study, with the advantage that the results can be stated in a clearer form. We also recall that the real and imaginary parts in (1) correspond to so-called even and odd cells

$$\begin{array}{rl} \psi_{q,p}^{\sigma }(x)= & \frac{1}{\sigma \sqrt{\pi }}\cos\left(p\cdot (x-q)\right)e^{-|x-q{|}^{2}/(2\sigma^{2})}\\ & +i\frac{1}{\sigma \sqrt{\pi }}\sin\left(p\cdot (x-q)\right)e^{-|x-q{|}^{2}/(2\sigma^{2})} \end{array}$$

but it will be sufficient for our purposes to deal with the full complex function as a whole.

### 2.1 Groups of Transformations

Let us introduce the following unitary operators on $L^{2}(R^{2})$: 

(i) translations: $T_{q}f(x)=f(x-q)$, $q\in R^{2}$,

(ii) modulations: $M_{p}f(x)=e^{ipx}f(x)$, $p\in R^{2}$,

(iii) dilations: $\Sigma_{\sigma }f(x)=\frac{1}{\sigma }f(\frac{x}{\sigma })$, $\sigma \in R^{+}$,

(iv) rotations: $R_{\alpha }f(x)=f(r_{-\alpha }x)$, $\alpha \in S^{2}$,

 where $r_{-\alpha }$ stands for the usual counterclockwise rotation of an angle *θ* on the Euclidean plane. In particular, we note that rotations commute with dilations, and it is easy to see that

(2) $$R_{\alpha }M_{p}=M_{r_{\alpha }p}R_{\alpha }.$$

If we denote with $g_{1}$, a $L^{2}(R^{2})$ normalized isotropic Gaussian with unit standard deviation

$$g_{1}(x)=\frac{1}{\sqrt{\pi }}e^{-|x{|}^{2}/2}$$

then we can characterize the functions (1) in terms of the operators (i), (ii), and (iii) as

$$\psi_{q,p}^{\sigma }(x)=T_{q}M_{p}\Sigma_{\sigma }g_{1}(x).$$

Such a family is the prototype of a so-called wave packet systems [17], and much is known about these structures [18,19]. 

In this work, we will deal with the localization properties of (1) with respect to translations and local rotations, i.e., making use of the symmetries (i) and (iv), since they constitute two fundamental symmetries related to the mechanisms of visual perception in V1 (see, e.g., [9] and references therein). 

Local rotations are defined by

(3) $$R_{\alpha }^{q}f(x)≐f\left({\left(r_{\alpha }^{q}\right)}^{-1}x\right)=T_{q}R_{\alpha }T_{-q}f(x),$$

where $r_{\alpha }^{q}x=r_{\alpha }(x-q)+q$ is a rotation of the Euclidean plane around point *q*, and with respect to these transformations we have the following.

**Lemma 2.1***Let*$\psi_{q,p}^{\sigma }$*be as in* (1). *Then*

(4) $$R_{\alpha }^{q}\psi_{q,p}^{\sigma }(x)=\psi_{q,r_{\alpha }p}^{\sigma }(x).$$

*Proof* Using (2) and the definition of local rotations (3), we get

$$R_{\alpha }^{q}\psi_{q,p}^{\sigma }(x)=T_{q}R_{\alpha }T_{-q}T_{q}M_{p}\Sigma_{\sigma }g_{1}(x)=T_{q}R_{\alpha }M_{p}\Sigma_{\sigma }g_{1}(x)=T_{q}M_{r_{\theta }p}R_{\alpha }\Sigma_{\sigma }g_{1}(x)$$

so (4) follows since rotations commute with dilations and $g_{1}$ is isotropic, i.e., $R_{\alpha }g_{1}(x)=g_{1}(x)$. □

Actually, the fact that $g_{1}$ is isotropic allows to write the whole family (1) in terms of all the operators (i) to (iv). Indeed, denoting with *θ* the polar angle of *p*, that means p=|p|(cosθ,sinθ), we can write (1) as

ψq,pσ(x)=TqRθM(|p|0)Σσg1(x),

where M(|p|0)f(x)=ei|p|x1f(x), so another way to characterize the system of functions (1) is to consider a family {gσ,|p|=M(|p|0)Σσg1(x),|p|,σ∈R+} and rotate and translate each of its members. The aim of next section is actually to deduce properties on the localization of $\psi_{q,p}^{\sigma }$ with respect to the parameters *q* and *θ*, expressed in terms of the parameters |p| and *σ*.

## 3 Measures of Uncertainty

In this section, we characterize the uncertainty associated to joint measurements of positions and local orientations in terms of the properties of the measurement devices, expressed by $L^{2}$ functions, and quantify such uncertainties for the case of receptive profiles.

We recall that the generators $P_{j}$ of translations along the Cartesian axis are given by partial derivatives

(5) $$P_{j}≐\frac{d}{dq_{j}}{|}_{q=0}T_{q}=\partial_{x_{j}},\;j=1,2$$

while the generator $J^{q}$ of a rotation around point *q* can be written in terms of the ordinary infinitesimal rotation operator $J=x_{2}\partial_{x_{1}}-x_{1}\partial_{x_{2}}$

(6) $$J^{q}≐\frac{d}{d\alpha }{|}_{\alpha =0}R_{\theta }^{q}=T_{q}JT_{-q}$$

and acts as the skew self-adjoint operator on $L^{2}(R^{2})$

$$J^{q}f(x)=\frac{d}{d\alpha }{|}_{\alpha =0}f\left(r_{\alpha }^{-1}(x-q)+q\right)=\left((x_{2}-q_{2})\partial_{x_{1}}-(x_{1}-q_{1})\partial_{x_{2}}\right)f(x).$$

We will measure averages and variances using the standard definitions for operators on $L^{2}$, denoting with ${\langle \cdot ,\cdot \rangle }_{L^{2}(R^{2})}$ the $L^{2}(R^{2})$ scalar product and with ${∥\cdot ∥}_{L^{2}(R^{2})}$ the associated norm.

**Definition 3.1** Let *L* be a densely defined skew self-adjoint linear operator on $D\subset L^{2}(R^{2})$. We define its mean value over f∈D as

(7) $$E_{f}(L)≐{\langle (iL)f,f\rangle }_{L^{2}(R^{2})}\in R$$

and its variance over f∈D as

(8) $${\left(\Delta_{f}L\right)}^{2}≐E_{f}\left({\left(L-E_{f}(L)\right)}^{2}\right)={∥\left(L-E_{f}(L)\right)f∥}_{L^{2}(R^{2})}^{2}.$$

Since skew self-adjoint operators are the infinitesimal generators of a one parameter group of unitary transformations, the meaning of the average (7) is that of measuring the deformation of *f* under such transformations

$$E_{f}(L)=i\lim_{t\to 0}\frac{{\langle (\exp tL)f,f\rangle }_{L^{2}(R^{2})}-{\langle f,f\rangle }_{L^{2}(R^{2})}}{t}$$

and the imaginary constant is merely a convention to ensure the result to be real. With this averaging, the variance (8) has the usual meaning of strength of the fluctuations of *f* under the considered transformations that corresponds to the second moment of the distribution $t\mapsto {\langle (\exp tL)f,f\rangle }_{L^{2}(R^{2})}$. This means then that the variance (8) provides a measure of the *localization* of *f* with respect to the symmetry exptL.

When applied to the operators (5) and (6) of linear and rotational derivatives, these variances correspond respectively to a measure of linear and rotational fluctuations of a function *f*. The more *f* is insensitive to translations (*f* smooth and close to a constant function), the smaller is its *P* variance, while a small $J^{q}$ variance means that *f* has little sensitivity to rotations around *q*.

The notion of *localization in orientation* that arises indicates that a function consisting of a set of parallel stripes, independently on their widths, is maximally localized in orientation, while a function that is circular symmetric around *q* is minimally localized.

If we are interested in the joint localization properties of a function with respect to a two parameters group of unitary transformations, generated by two skew self-adjoint operators $L_{1}$ and $L_{2}$, we are led to consider the distribution

(9) $$(t_{1},t_{2})\mapsto {\langle (\exp t_{1}L_{1})(\exp t_{2}L_{2})f,f\rangle }_{L^{2}(R^{2})}.$$

In this case, if the operators $L_{1}$ and $L_{2}$ do not commute, then the second moments of the distribution (9) are influenced by their commutator. Such an effect of competing symmetries is quantified by the uncertainty principle.

### 3.1 The SE(2) Uncertainty Principle

The operators (5) and (6) satisfy the commutation relations of angular momentum [20]

(10) $$\left[J^{q},P_{1}\right]=P_{2};\;\left[J^{q},P_{2}\right]=-P_{1}.$$

These commutators define the algebra of the SE(2) group (see, e.g., [9] and references therein), and for them the following generalized uncertainty principle holds [13,21], with respect to the quantities of Definition 3.1. Since we are dealing with densely defined operators, we will skip in what follows the technicalities related to operator domains, and refer the statements simply to $L^{2}(R^{2})$. For more details, see [21]. 

**Theorem 3.2** (SE(2) uncertainty principle)

*For any*$f\in L^{2}(R^{2})$, *it holds*

(11) $$\left\{ \begin{array}{c} (\Delta_{f}J^{q})(\Delta_{f}P_{1})\geq \frac{1}{2}|E_{f}(P_{2})|,\\ (\Delta_{f}J^{q})(\Delta_{f}P_{2})\geq \frac{1}{2}|E_{f}(P_{1})|. \end{array}\right.$$

These inequalities play the same role for the noncommutative symmetries of rotations and translations as the one played by the ordinary uncertainty inequality for the noncommutativity of quantum mechanical operators. The main difference is that in this case if we consider separately each of the two inequalities, we cannot obtain a constant lower bound. Indeed for a function *f* the product of variances of an infinitesimal rotations and a translations along one axis can be arbitrarily small, provided that the average of translations along the other axis on *f* is small. This effect disappears when we consider translations on both axis, which is natural whenever we do not want to discriminate one direction over the other. In this case, we can actually recast the two inequalities (11) into one inequality with a constant lower bound.

The following definition is closely related to that of [22], and for this reason we use the same notation Angv. 

**Definition 3.3** Let us define the functional

$$\mathrm{Angv}[f]≐\frac{\Delta_{f}P}{E_{f}(P)},$$

where

$$E_{f}(P)≐\sqrt{{\left(E_{f}(P_{1})\right)}^{2}+{\left(E_{f}(P_{2})\right)}^{2}}\;\text{and}\;\Delta_{f}P≐\sqrt{{\left(\Delta_{f}P_{1}\right)}^{2}+{\left(\Delta_{f}P_{2}\right)}^{2}}.$$

We denote with ΔΘ[f] the corresponding measure of angular uncertainty

(12) ΔΘ[f]≐arctan(Angv[f]).

With this definition, a direct consequence of the SE(2) uncertainty principle is the following.

**Theorem 3.4***For all*$f\in L^{2}(R^{2})$

(13) $$\left(\Delta_{f}J^{q}\right)\mathrm{Angv}[f]\geq \frac{1}{2}.$$

This inequality resembles the ordinary Heisenberg uncertainty inequality, since the presence of a constant lower bound provides a clear constraint on the joint localizations quantified by $\Delta_{f}J^{q}$ and Angv[f]. However, as first noted in [23], the SE(2) uncertainty inequalities (11) cannot be simultaneously minimized, so also (13) does not admit minimizers. This is related to the issue of nonexistence of a canonically conjugate observable for angular momentum [24,25]. Indeed, if we had a well defined self-adjoint operator canonically commuting with angular momentum, we would end up with a well-known complex equation defining minimal uncertainty states [21], while in this case we have two such equations, whose solutions provide CR function functions on the $R^{2}\times S^{1}$ for two noncompatible almost complex structures [26]. 

### 3.2 SE(2) Autocorrelations

We pass now to the study of the properties of the distribution (9) applied to the symmetries under study, that we call autocorrelation since it has the form of the autocorrelation of a function with respect to the group of rotations and translations, and extends naturally the ordinary definition of autocorrelation with respect to translations. We will actually restrict the analysis to the square modulus of correlations, since as we will see it contains enough information for the present purposes. In particular, we will show that such correlations can be used to characterize the uncertainty in the detection of position and local preferred angle associated to a function.

**Definition 3.5** Given *f* in $L^{2}(R^{2})$, we define its SE(2) autocorrelation centered at *q* as

(14) $$C_{q}[f](\xi ,\alpha )=|{\langle T_{\xi }R_{\alpha }^{q}f,f\rangle }_{L^{2}(R^{2})}{|}^{2}.$$

In general, $C_{q}[f](\xi ,\alpha )$ provides a natural way to study the joint localization properties of *f* with respect to position and local preferred angle. Indeed, when we specialize to translations we get the usual autocorrelation, and by Plancherel theorem

(15) $$C_{q}[f](\xi ,0)=|\int_{R^{2}}f(x-\xi )\bar{f(x)}dx{|}^{2}=|F\left(|Ff{|}^{2}\right)(\xi ){|}^{2}$$

so we have that by Young inequality and the Riemann–Lebesgue lemma $C_{q}[f](\xi ,0)$ is bounded and goes to 0 as *ξ* becomes large. Moreover, by the usual uncertainty principle, we have that when *f* is well localized in space, then Ff is broadly localized, hence passing under another Fourier transform $C_{q}[f](\xi ,0)$ will decay rapidly, uniformly on *q*, and vice versa.

On the other hand, if we consider correlations only with respect to rotations, for simplicity centered at q=0

(16) $$C_{0}[f](0,\alpha )=|\int_{R^{2}}f(r_{-\alpha }x)\bar{f(x)}dx{|}^{2}$$

essentially the same argument applies to the decay of correlations for functions that are localized with respect to rotations.

**Remark 3.6** (What does “essentially the same argument” mean)

Since $FR_{\theta }f=R_{\theta }Ff$, we get

$$C_{0}[f](0,\alpha )=|\int_{R^{2}}f(r_{-\alpha }x)\bar{f(x)}dx{|}^{2}=|\int_{R^{2}}R_{\alpha }(Ff)(k)\bar{Ff(k)}dk{|}^{2}$$

so setting polar coordinates, with the notation $\phi_{\kappa }(\varphi )=(Ff)(\kappa \cos\varphi ,\kappa \sin\varphi )$

$$C_{0}[f](0,\alpha )=\int_{R^{+}}\kappa d\kappa \int_{0}^{2\pi }\phi_{\kappa }(\varphi -\alpha )\bar{\phi_{\kappa }(\varphi )}=\int_{R^{+}}\kappa d\kappa \sum_{n\in Z}e^{-2\pi in\alpha }|{\hat{\phi }}_{\kappa }(n){|}^{2},$$

where ${\hat{\phi }}_{\kappa }(n)=\int_{0}^{2\pi }e^{-2\pi in\varphi }\phi_{\kappa }(\varphi )d\varphi$ and the last transition is Parseval identity.

Since $L^{2}(R^{2})\approx L^{2}(R^{+},\kappa d\kappa )⊗L^{2}(S^{1})$ as a tensor product of Hilbert spaces, and since *f* is localized with respect to rotations in the real plane if and only if it is localized with respect to rotations in the Fourier plane, then we can assume without loss of generality that $\phi_{\kappa }(\varphi )=r(\kappa )\Phi (\varphi )$, where Φ(φ) decays rapidly away from φ=0 and $\int_{R^{+}}{\left|r(\kappa )\right|}^{2}\kappa d\kappa =c<\infty$. So,

$$C_{0}[f](0,\alpha )=c\sum_{n\in Z}e^{-2\pi in\alpha }|\hat{\Phi }(n){|}^{2}$$

and now strictly the same argument used for (15) applies.

### 3.3 Uncertainty Associated to SE(2) Measurements with Receptive Profiles

When specialized to receptive profiles, the introduced uncertainties can be explicitly computed. In the proofs, we will use the shorthand notation $g_{\sigma }=\Sigma_{\sigma }g_{1}$, and *θ* will be the polar angle of *p*.

**Lemma 3.7***The variance of the operators* (5) *on receptive profiles* (1) *is*

$$\Delta_{\psi_{qp}^{\sigma }}P_{j}=\frac{1}{\sqrt{2}\sigma }.$$

*Proof* Since $\partial_{x_{j}}\psi_{qp}^{\sigma }(x)=(ip_{j}-\frac{\left(x_{j}-q_{j}\right)}{\sigma^{2}})\psi_{qp}^{\sigma }(x)$, we get $E_{\psi_{qp}^{\sigma }}(P_{j})=ip_{j}$, and

$$\begin{array}{rcl} E_{\psi_{qp}^{\sigma }}\left(P_{j}^{2}\right) & = & {∥P_{j}\psi_{qp}^{\sigma }∥}_{L^{2}(R^{2})}^{2}=\int_{R^{2}}|p_{j}+i\frac{\left(x_{j}-q_{j}\right)}{\sigma^{2}}{|}^{2}|g_{\sigma }(x-q){|}^{2}dx\\ & = & p_{j}^{2}+\frac{1}{\pi \sigma^{2}}\int_{R^{2}}y_{j}^{2}e^{-y^{2}}dy=p_{j}^{2}+\frac{1}{2\sigma^{2}}. \end{array}$$

 □

**Lemma 3.8***The variance of the operator* (6) *on receptive profiles* (1) *is*

(17) $$\Delta J≐\Delta_{\psi_{qp}^{\sigma }}J^{q}=\frac{|p|\sigma }{\sqrt{2}}$$

*and we will call it angular momentum variance*.

*Proof* Since

$$\partial_{x_{j}}\psi_{qp}^{\sigma }(x)=\left(ip_{j}-\frac{\left(x_{j}-q_{j}\right)}{\sigma^{2}}\right)\psi_{qp}^{\sigma }(x)$$

then

$$J^{q}\psi_{qp}^{\sigma }(x)=i\left((x_{2}-q_{2})p_{1}-(x_{1}-q_{1})p_{2}\right)\psi_{qp}^{\sigma }(x).$$

Its mean value vanishes on $\psi_{qp}^{\sigma }$, due to the isotropy of $g_{\sigma }$:

$$\begin{array}{rcl} E_{\psi_{qp}^{\sigma }}\left(J^{q}\right) & = & {\langle J^{q}\psi_{qp}^{\sigma },\psi_{qp}^{\sigma }\rangle }_{L^{2}(R^{2})}=i\int_{R^{2}}(x_{2}p_{1}-x_{1}p_{2})|g_{\sigma }(x){|}^{2}dx\\ & = & i|p|\int_{R^{2}}{\left(r_{-\theta }x\right)}_{2}|g_{\sigma }(x){|}^{2}dx=i|p|\int_{R^{2}}x_{2}|g_{\sigma }(x){|}^{2}dx=0. \end{array}$$

To compute the variance, by analogous arguments

$$\begin{array}{rcl} {\left(\Delta_{\psi_{qp}^{\sigma }}J^{q}\right)}^{2} & = & -|p{|}^{2}\int_{R^{2}}{\left({\left(r_{-\theta }x\right)}_{2}\right)}^{2}|g_{\sigma }(x){|}^{2}dx\\ & = & -\frac{|p{|}^{2}}{{\left(\sigma \sqrt{\pi }\right)}^{2}}\left(\int_{R}e^{-x_{1}^{2}/\sigma^{2}}dx_{1}\right)\left(\int_{R}x_{2}^{2}e^{-x_{2}^{2}/\sigma^{2}}dx_{2}\right)\\ & = & \frac{|p{|}^{2}\sigma^{2}}{2\sqrt{\pi }}\int_{R}y\left(-2ye^{-y^{2}}\right)dy={\left(\frac{|p|\sigma }{\sqrt{2}}\right)}^{2}. \end{array}$$

 □

We have then obtained the following proposition, which shows that for receptive profiles the angular momentum variance ΔJ is inversely proportional to the angular uncertainty quantified in terms of Angv.

**Proposition 3.9***Let*$\psi_{qp}^{\sigma }$*be as in* (1). *Then*

$$\Delta J\mathrm{Angv}\left[\psi_{qp}^{\sigma }\right]=\frac{1}{\sqrt{2}}.$$

*Proof* Using Definition 3.3, Lemma 3.7, and Lemma 3.8, we have

(18) $$(\mathrm{Angv})\left[\psi_{qp}^{\sigma }\right]=\frac{1}{\sigma |p|}=\frac{1}{\sqrt{2}\Delta J}.$$

 □

We will now consider SE(2) autocorrelations of receptive profiles, and see that they indeed contain precisely the desired joint information on localizations in space and local orientation associated to the uncertainties we computed.

**Proposition 3.10***Let*$\psi_{qp}^{\sigma }$*be defined by* (1). *Then its*SE(2)-*autocorrelation reads*

(19) $$C_{q}\left[\psi_{qp}^{\sigma }\right](\xi ,\alpha )=e^{-|\xi {|}^{2}/(2\sigma^{2})}e^{-{\left(\Delta J\right)}^{2}(1-\cos\alpha )}.$$

*Proof* By Lemma 2.1, and computing the Fourier transform of a Gaussian

$$\begin{array}{rcl} {\langle T_{\xi }R_{\alpha }^{q}\psi_{q,p}^{\sigma },\psi_{q,p}^{\sigma }\rangle }_{L^{2}(R^{2})} & = & {\langle T_{\xi }\psi_{0,r_{\alpha }p}^{\sigma },\psi_{0,p}^{\sigma }\rangle }_{L^{2}(R^{2})}\\ & = & \frac{1}{\sigma^{2}\pi }\int_{R^{2}}e^{i(r_{\alpha }p)(x-\xi )}e^{-|x-\xi {|}^{2}/(2\sigma^{2})}e^{-ipx}e^{-|x{|}^{2}/(2\sigma^{2})}dx\\ & = & \frac{e^{-i(r_{\alpha }p)\xi }}{\sigma^{2}\pi }e^{-|\xi {|}^{2}/(4\sigma^{2})}\int_{R^{2}}e^{-i(p-(r_{\alpha }p))x}e^{-|x-\xi /2{|}^{2}/(\sigma^{2})}dx\\ & = & e^{-i((p+(r_{\alpha }p))/2)\xi }e^{-|\xi {|}^{2}/(4\sigma^{2})}e^{-\sigma^{2}|p-(r_{\alpha }p){|}^{2}/4} \end{array}$$

so the result follows since $\left|p-(r_{\alpha }p){|}^{2}=2|p{|}^{2}(1-\cos\alpha \right)$. □

This proposition shows that the decay of the autocorrelation in space is a Gaussian with the same width of the corresponding receptive profile that characterize spatial uncertainty. With respect to rotations, we have ended up with a Von Mises distribution in orientations. Such distributions appear naturally when discussing the SE(2) uncertainty principle [20,27], but they also provide a good model for orientation tuning of simple cells [28], which is defined as the response curve of a cell to oriented stimuli [28,29]. This confirms that the introduced notion of localization is compatible with the resolution of measurements performed with receptive profiles. Moreover, we note that the commonly used circular variance [30] of the Von Mises distribution in (19), up to a normalization constant, is

$$\mathrm{CircVar}(\Delta J)=1-\frac{I_{1}({\left(\Delta J\right)}^{2})}{I_{0}({\left(\Delta J\right)}^{2})}$$

which results to be numerically close to what we have introduced as angular uncertainty (12) when applied to receptive profiles (18)

$${\left(\Delta \Theta \left[\psi_{q,p}^{\sigma }\right]\right)}^{2}={\left(\arctan\left(\frac{1}{\sqrt{2}\Delta J}\right)\right)}^{2}.$$

In particular, as we will see in next section, typical values of ΔJ in the filters encountered in V1 are around 1.7, where the difference between these two notions of variance is around $5\cdot 10^{-2}$.

## 4 Bounds on the Shape Index Induced by Uncertainty

In this section, we will use the measures of uncertainty referred to receptive profiles (18) and (19) to deduce relevant features about the physiological data measured in [3] and depicted in Fig. 1. In particular, we will see how the information provided by the analysis of uncertainty relations of Sect. 3 are sufficient to establish bounds on the number of subregions observed in the family of filters implemented in V1, and permit to reobtain characteristic sampling rates commonly used in image analysis.

A receptive profile $\psi_{qp}^{\sigma }$ consists of an oscillation of frequency $\nu =\frac{\left|p\right|}{2\pi }$ under a Gaussian bell of width *σ*, so it appears natural to define a dimensionless index of shape [3]

(20) n=νσ.

This quantity is related to the number of subregions defining a receptive profiles, since if we let $N_{k}$ be the number of half wavelength of receptive profile’s oscillation within *k* standard deviations *σ*, we obtain $N_{k}=4kn$. As it is apparent from the data measured in [3], we see that approximately k=2 standard deviations are sufficient to represent the main content of the filters, so that we can relate the effective subregions *N* to *n* as N=8n.

In terms of *n*, the angular momentum variance (17) of a receptive profile $\psi_{qp}$ reads

(21) $$\Delta J=\sqrt{2}\pi n$$

while its angular variance (12), after (18), reads

(22) $$\Delta \Theta \left[\psi_{qp}^{\sigma }\right]=\arctan\left(\frac{1}{2\pi n}\right).$$

### 4.1 Lower Bound for Orientation Measurements

By the discussion in Sect. 3, we have seen how we can quantify with *ΔΘ* the angle resolution allowed by a linear filtering. If we refer to the task of orientation detection, we can set as a reasonable bound that of angle uncertainty less than π/2, that is expressed by

$$\Delta \Theta \left[\psi_{qp}^{\sigma }\right]\leq \frac{\pi }{4}.$$

This condition can be stated in terms of the shape index using (22)

$$n\geq \frac{1}{2\pi }\approx 0.16≐n_{\min}.$$

As we can see in Fig. 1 and by the discussions in [3], cells which show a selectivity in orientation all lie above this threshold. Moreover, we note that for indexes $n<n_{\min}$, it can be a hard task to distinguish an even cell from being represented only by a Gaussian, while odd cells under this threshold all appear identical up to a multiplicative factor, so the parametric fit of the Gabor model (1) is quite delicate in this region. We can then interpret the bunch of broadly tuned cells around the zero value of the shape index *n* as generally below the minimal uncertainty bound that allows a consistent detection of orientations.

### 4.2 Upper Bound

In order to discuss the upper bound, we introduce a notion of characteristic length associated to a specific level set of the correlations (1), intrinsically related to the task of detection of positions and local orientations. Its purpose is to quantify the minimum distance that one needs to cover in order to decorrelate a function *f* as much as *f* is decorrelated when compared at orthogonal directions.

**Definition 4.1** The correlation length for $f\in L^{2}(R^{2})$ is the smallest distance *λ* for which

(23) $$C[f](\xi ,0)\leq C[f]\left(0,\frac{\pi }{2}\right)\;\forall |\xi |=\lambda .$$

If we apply this notion to receptive profiles (1), we obtain the following.

**Proposition 4.2***The shape index* (20) *is bounded from above by the ratio of the correlation lengthλand the spatial uncertaintyσ*

(24) $$2\pi n\leq \frac{\lambda }{\sigma }.$$

*Proof* Condition (23) on receptive profiles $\psi_{qp}^{\sigma }$, by (19) reads $\Delta J\leq \frac{\sqrt{2}}{2}\frac{\lambda }{\sigma }$, since

$$e^{-\lambda^{2}/(2\sigma^{2})}\leq e^{-\Delta J^{2}}\;\Leftrightarrow \;\frac{\lambda^{2}}{2\sigma^{2}}\geq \Delta J^{2}$$

so (24) follows by the relation (21) between ΔJ and the shape index. □

On the other hand, as discussed when dealing with the relation between the shape index and the number of subregions, we have also that the effective field of influence of a receptive profile can be set within two standard deviations *σ*. From this point of view, we can then assume that the distance *d* at which a receptive profile is effectively spatially uncorrelated corresponds to the distance that one has to cover in order to let its effective field of influence not intersect with its translation at a distance *d*, i.e., d=4σ.

In order to couple with both position and orientation measurements, we will then consider the hypothesis of balance of the two characteristic scales introduced that is the identification λ=d. By (24), this condition can be stated in terms of the shape index as

$$n\leq \frac{2}{\pi }\approx 0.64≐n_{\max}.$$

To compare this bound with Fig. 1, we recall that here we are dealing with the simplified model of isotropic receptive fields, while in [3] the analysis is performed considering two anisotropic indexes $n_{x}$ and $n_{y}$. In terms of such indexes, we can see that the largest part of the population lies within two bounds $n_{x}≲0.5$ and $n_{y}≲0.76$, and $n_{\max}$ looks in good accordance with their mean value.

The question of whether this identification of characteristic distances is truly implemented in the cortex cannot be answered at this point, but we note that a cortical scale related to the symmetries under study that is possibly compatible with the proposed relation is the mean correlation length of orientation preference maps (see, e.g., [13,14] and references therein). Indeed, by the measurements performed in [31], we see that such scale is comparable with the size of a so-called cortical point image, that is, the cortical region that is activated after a highly spatially localized stimulus, and at least when we reduce to linear behavior of cells this notion corresponds to what we have indicated as effective field of influence. 

### 4.3 Sampling on Orientations

Another intriguing consequence of the performed uncertainty analysis can be stated in terms of optimal sampling rates for orientation detection. Indeed, if we consider the mean value on shape index measured in [3], or equivalently, in terms of the deduced bounds, for $n=\frac{n_{\max}+n_{\min}}{2}\approx 0.4$, we have that

$$\Delta \Theta \left[\psi_{qp}^{\sigma }\right]=\arctan\left(\frac{1}{0.8\pi }\right)\approx \frac{\pi }{8}.$$

With respect to Gabor filters possessing such *n*, one way to use such result is to consider that the detection of orientations at angles that are closer than this uncertainty do not provide an actual improvement in the resolution of the local orientation present in the stimulus, so that it can be sufficient to cover the interval of orientations [0,π) with a sampling having a π/8 spacing. This actually compares well with the notions of optimal sampling adopted in image analysis tasks (see, e.g., in [32] and references therein), generally justified with independent arguments. Moreover, this uncertainty analysis permits to set clear sampling spacings depending on the shape index of the filter used. 

## 5 Conclusions

In this paper, we have studied theoretical aspects of an analytic characterization of uncertainty that generalizes the well-known Heisenberg uncertainty principle to the symmetries associated with the task of joint measurements of position and local orientation. The implications of this analysis, together with an hypothesis of balance between characteristic correlation distances, allowed us to obtain bounds comparable with experimental data on the shape index of the V1 simple cells that are selective for orientation, and to separate them from broadly tuned cells, which lie below the uncertainty bound for consistent orientation detection.

We remark that this was possible even if our working assumptions on the functional behavior of simple cells were reduced to linear filtering with symmetric receptive fields, and the only considered task is the one associated to the sole symmetries of rotations and translations.

Whether such elementary principles could be directly responsible of the observed distribution of receptive profiles is a question that can hardly find an answer. Nevertheless, the present study shows that they are sufficient to describe many of the relevant features that concern the shape of simple cells.

## Competing Interests

The authors declare that they have no competing interests.

## Authors’ Contributions

All authors contributed equally to the writing of this paper. All authors read and approved the final manuscript.
